# The Toxic Effects of Petroleum Diesel, Biodiesel, and Renewable Diesel Exhaust Particles on Human Alveolar Epithelial Cells

**DOI:** 10.3390/jox14040080

**Published:** 2024-10-09

**Authors:** Oskari J. Uski, Gregory Rankin, Håkan Wingfors, Roger Magnusson, Christoffer Boman, Robert Lindgren, Ala Muala, Anders Blomberg, Jenny A. Bosson, Thomas Sandström

**Affiliations:** 1 Department of Public Health and Clinical Medicine, Umeå University, 90187 Umeå, Sweden; gregory.rankin@foi.se (G.R.);; 2 CBRN Defence and Security, Swedish Defence Research Agency, 90182 Umeå, Sweden; 3 Department of Applied Physics and Electronics, Thermochemical Energy Conversion Laboratory, Umeå University, 90187 Umeå, Sweden

**Keywords:** biodiesel, renewable diesel, petrodiesel, rapeseed methyl ester, soy methyl ester, hydrotreated vegetable oil, particulate matter, emissions, in vitro toxicology

## Abstract

The use of alternative diesel fuels has increased due to the demand for renewable energy sources. There is limited knowledge regarding the potential health effects caused by exhaust emissions from biodiesel- and renewable diesel-fueled engines. This study investigates the toxic effects of particulate matter (PM) emissions from a diesel engine powered by conventional petroleum diesel fuel (SD10) and two biodiesel and renewable diesel fuels in vitro. The fuels used were rapeseed methyl ester (RME), soy methyl ester (SME), and Hydrogenated Vegetable Oil (HVO), either pure or as 50% blends with SD10. Additionally, a 5% RME blend was also used. The highest concentration of polycyclic aromatic hydrocarbon emissions and elemental carbon (EC) was found in conventional diesel and the 5% RME blend. HVO PM samples also exhibited a high amount of EC. A dose-dependent genotoxic response was detected with PM from SD10, pure SME, and RME as well as their blends. Reactive oxygen species levels were several times higher in cells exposed to PM from SD10, pure HVO, and especially the 5% RME blend. Apoptotic cell death was observed in cells exposed to PM from SD10, 5% RME blend, the 50% SME blend, and HVO samples. In conclusion, all diesel PM samples, including biodiesel and renewable diesel fuels, exhibited toxicity.

## 1. Introduction

The combustion of fossil fuels has increased the threat of global warming [1] and has thus created a demand for more carbon-neutral and renewable fuels for use in commuter traffic and personal transport [2]. Diesel engines are among the main sources of particulate matter (PM) in urban areas [3]. Diesel exhaust particles (DEPs) cause adverse health effects in humans, such as the development or worsening of respiratory illnesses, including asthma [4]. In addition, exposure to DEPs has been linked to an increased risk of atherosclerosis, hypertension, and cancer development [5,6,7]. More recently, neurodegenerative disorders have been connected to DEPs exposure [8]. With limited knowledge of the potential negative health effects of exposure to biodiesel and renewable diesel fuel emissions in humans, there is an urgent need to elucidate their short- and long-term health effects.

Biodiesel and renewable diesel produced for existing diesel engines typically originate from vegetable oils and other fatty acids and are formed either by transesterification to produce suitable fatty acid methyl esters (FAMEs) or by thermal hydrodeoxygenation to produce Hydrogenated Vegetable Oil (HVO) [9]. The chemical composition and properties of biodiesels and renewable diesel depend on the production method. FAME biodiesel is enriched with oxygen in comparison with HVO and conventional diesel. Moreover, FAMEs and HVO lack sulfur and contain fewer aromatic hydrocarbons (HCs) than conventional diesel fuel [9].

It is known that engine combustion of biodiesel and renewable diesel usually produces less PM, carbon monoxide, and total HCs than conventional diesel fuels [10]. However, there is a strong suggestion that biodiesel and renewable diesel combustion produce emissions that differ considerably from traditional diesel fuels [11,12], which may cause unexpected adverse health impacts [11,13,14]. Emissions from biodiesel and renewable diesel differ from petroleum diesel by their content of PM-bound organic carbon and a higher count of nano-sized particles. Indeed, biodiesel and renewable diesel usually produce lower polycyclic aromatic hydrocarbon (PAH) and soot emissions but generate smaller PM [15,16]. In addition, biodiesel and renewable diesel fuel have a higher oxygen content, which may lead to more reactive semi-volatile species and PM with greater oxidative reactivity [16].

When biodiesel, renewable diesel, and traditional diesel engine combustion-derived PM is evaluated for toxicity, the study outcomes show considerable diversity [17,18]. For instance, some studies have reported that biodiesel and renewable diesel PM is more toxic than PM from conventional petroleum diesel [19,20,21,22]. Conversely, many studies suggest that biodiesel- and renewable diesel-derived PM is comparable to or less toxic than conventional diesel PM [23,24,25,26]. This disparity in the literature regarding the health effects of biodiesel and renewable diesel PM highlights the need for well-controlled toxicological studies.

The main purpose of this study was to characterize diesel emission particles from both chemical and toxicological perspectives. The current study used a heavy-duty diesel engine under the urban part of the European Transient Cycle (ETC) to generate PM. This study design was selected to mimic the PM to which humans are commonly exposed in large amounts, both in terms of PM concentration and population exposure [27]. Moreover, to collect PM, we employed the rarely used DEPs PM collection method, which better mimics PM deposition in human lung and captures the chemistry and structure of PM more effectively than traditional methods based on inert impaction [28]. PM was derived from a diesel engine powered by a European standardized reference low-sulfur petroleum diesel fuel (SD10, RF-06-03), two different biodiesel fuels, and one renewable diesel fuel, along with their blends with SD10. The biodiesel fuels (FAMEs) were rapeseed methyl ester (RME) and soy methyl ester (SME), which represent common biofuels in regions such as Europe and North America. The renewable diesel was a second-generation biofuel, Hydrogenated Vegetable Oil (HVO), which is also already used in Europe and North America. The fuels were tested in the following combinations: SD10, RME, SME, and HVO in their pure forms (100%) as well as 50% blends with SD10. Moreover, RME was also tested as a 5% blend with 95% SD10. The blends selected for the current study were based on needs to replace pure petrodiesel fuels as much as possible, preferably with 100% renewable fuels, while addressing concerns for some engine types and generations that lower blends may be needed [29]. Since RME has been widely established in parts of Europe as a 5% blend, that alternative was also included [30]. To enhance the real-world relevance of this study, an extensive chemical analysis of PM was conducted, covering PAHs, oxygenated polycyclic aromatic hydrocarbons (Oxy-PAHs), organic carbon (OC), elemental carbon (EC), and metals. Additionally, toxicological endpoints like cytotoxicity, genotoxicity, oxidative stress, and inflammation were studied using the human alveolar basal epithelial cell line (A549). This comprehensive approach provides a detailed assessment of the toxicological effects of real-life relevant biofuels.

## 2. Materials and Methods

### 2.1. Engine, Exhaust Dilution, and Fuels

Exhaust emissions from various diesel fuels were produced using a heavy-duty diesel engine (Volvo TD45, 4.5 L, 4-cylinder, direct injection, manufactured in 1991), compliant with EU Stage I and Tier 1 particle emission standards (0.54 g/kW h PM). The engine was connected to an engine dynamometer. The diesel engine was operated using the ETC urban part, as described previously [31]. This system for exhaust generation has been validated and used in several experimental human studies [24,32].

Four different low-sulfur fuels were included in the present study. A reference petroleum Standard Diesel fuel (SD10) and the two biofuels, RME and HVO, were delivered by Preem (Preem AB, Stockholm, Sweden). The biofuel SME was delivered from Coryton (The Manorway, Coryton, Stanford Le Hope, Essex, UK). The fuels were tested in the following combinations: SD10, RME, SME, and HVO as pure fuels (100%) and as 50% blends with the standard petrodiesel, SD10. Moreover, RME was also tested as a 5% blend with 95% SD10. The generation of exhaust using SD10 was repeated on three different occasions and all 100% biofuels on two different occasions. A representative aliquot of the unblended fuels was taken from the fuel delivery system in the middle of each PM sampling period and analyzed for PAHs and Oxy-PAHs compounds.

### 2.2. Sampling and Chemical/Physical Characterization of DEPs

Diluted exhaust was sampled from the dilution tunnel after the first dilution step with filtered air (see figure in Supplementary File S1). For in vitro testing, DEPs were collected using a custom-built system comprising an aerosol condensational growth unit and bioaerosol impingers (BioSampler^®^, SKC Inc., Eighty-Four, PA, USA). Sampling systems of this nature have demonstrated the ability to effectively collect both ultrafine and fine particles. When these aerosols are concentrated in a biocompatible liquid medium, their physical and chemical properties are largely preserved [33,34,35,36]. Supplementary File S1 includes a sketch and further details of the system for collection of DEPs for subsequent chemical characterization and toxicological analysis. See also Uski et al. [28] for more detailed description of the collection method and the sample handling.

Particle mass concentration in the diluted exhaust flow was determined from DEPs mass collected on polytetrafluoroethylene (PTFE) filters before the aerosol condensational growth unit (see Supplementary File S1). The content of 44 PAHs and 12 Oxy-PAHs in DEPs was determined after extraction, cleanup, and analysis of the PTFE filters according to a previously published protocol with some minor modifications [37]. Metal content of DEPs was determined by analyzing the PTFE filter samples, while the fraction of OC and EC was assessed by thermal-optical analyses of quartz-filter samples [38,39] (see Supplementary File S2 for more details).

A differential mobility particle sizer (DMPS), consisting of an electrostatic classifier (model 3071, TSI Inc., Shoreview, MN, USA) and a condensation particle counter (CPC, model 3010, TSI Inc.), was used to measure particle number size distribution of the sub-micron (<1 µm) fraction in diluted exhaust after the second dilution step.

### 2.3. Sample Preparation for Cell Studies

To prepare the particulate bioaerosol impinger stock samples for cell experiments, they were first sonicated for 30 min in an ultrasonic water bath. The samples were then portioned into aliquots and kept at −20 °C until use. Before using them for in vitro experiments, the aliquots were thawed at room temperature for 30 min and subsequently sonicated for another 30 min in an ultrasonic water bath before cell exposure. A more detailed sample preparation procedure is described by Uski et al. [28].

### 2.4. Experimental Setup for Toxicological Study

A549 cells (ATCC, Rockville, MD, USA) were cultured in Dulbecco’s Modified Eagle Medium (DMEM) supplemented with 10% fetal bovine serum (FBS), 2 mM L-glutamine, and 100 U/mL penicillin/streptomycin (all from Sigma-Aldrich Corp., St. Louis, MO, USA). Cells were maintained in a humidified incubator at 37 °C with 5% CO_2_. For subculturing or when dividing cells in 12-well plates, the cell layer was washed with Dulbecco’s phosphate buffered saline (D-PBS) (Sigma-Aldrich Corp., St. Louis, MO, USA) before the addition of 2 mL Trypsin-EDTA solution (Sigma-Aldrich Corp., St. Louis, MO, USA) to detach the cells. The cells were subsequently counted and seeded at a density of 150,000 cells per well in 12-well plates for the exposure experiments. Following a 24 h attachment phase, the culture medium was replaced, and the cells were allowed to acclimate for one hour. During this acclimatization period, PM samples were prepared. Cells were exposed to four increasing concentrations of PM: 30 µg/mL, 75 µg/mL, 150 µg/mL, and 220 µg/mL for 24 h at 37 °C and 5% CO_2_ in a humidified incubator. Each experiment included untreated control (UN), water controls (adding 83 μL of W1503 water from Sigma-Aldrich Corp., St. Louis, MO, USA), and suitable positive controls for the toxicity assay.

After the 24 h exposure duration, the culture medium was gathered and stored at −80 °C for later cytokine examination of IL-6 and IL-8. The cells were rinsed with D-PBS (Sigma-Aldrich Corp., St. Louis, MO, USA) and detached from the well bottoms using trypsinization. Trypsin activity was halted by adding FBS. Aliquots of the cell suspension were taken for MTT analysis and detection of cellular reactive oxygen species (ROS). The remaining cells were used to determine DNA damage by comet assay and for cell cycle analysis.

### 2.5. Assessments of Inflammation Mediators

The levels of pro-inflammatory cytokines, interleukin (IL)-6 and IL-8, were analyzed using enzyme-linked immunosorbent assay (ELISA) kit (R&D Systems, Minneapolis, MN, USA) on 96-well plates (Nunc Maxisorp), according to the manufacturer’s instructions with the following exceptions: For the cytokine measurements, the sample size used for statistical analyses and figures was 5 (*n* = 5). Tumor necrosis factor alpha (TNF-α), at a concentration of 5 ng/mL, was employed as a positive control for cytokine analyses to ensure methodological reliability.

### 2.6. Analyses of Cell Viability

Cell viability was detected using the MTT test in 96-well plates. The test detects cells with functioning mitochondria and endoplasmic reticulum, as previously described [40]. Absorbance was measured at a wavelength of 570 nm using a multi-label plate reader, and cell viability was calculated as a percentage based on the readings from the control cells. In the MTT experiments, the sample size used for statistical analyses and figures was 5 (*n* = 5). The potential interference of the particulate samples with the method was also assessed and ruled out. Hydrogen peroxide, at a concentration of 13 mM, was used as a positive control.

### 2.7. Assessment of ROS inside Cells

Detection of cellular ROS was carried out in cell suspension aliquots. Cells were centrifuged at 6082 RCF for 5 min at 4 °C, the supernatant was discarded, and the cell pellet was resuspended in 220 µL D-PBS (Sigma-Aldrich Corp., St. Louis, MO, USA). The cells were then plated as 2 × 100 µL aliquots in 96-well plates, and 8 µL of 2′,7′-dichlorodihydrofluorescein diacetate (H2DCFDA) solution (0.5 µM in dimethyl sulfoxide) was added. The cells were incubated for 30 min at 37 °C before measuring the dichlorofluorescein fluorescence at 485 nm excitation and 530 nm emission. In the ROS experiments, the sample size used for statistical analyses and figures ranged from 4 to 5 (*n* = 4–5), depending on PM sample. Hydrogen peroxide (13 mM) was used as a positive control for the H2DCFDA assay.

### 2.8. Sub-G1 and Cell Cycle

Cell cycle phase distribution was analyzed from cells fixed in 70% ethanol. The cells were centrifuged (10 min, 400× *g*, +4 °C), and ethanol was removed. Cell pellets were washed with 1 mL of cold PBS and then resuspended in 1000 μL of cold PBS. RNAse A (0.15 mg/mL) (all Sigma-Aldrich Corp., St. Louis, MO, USA) was added to each tube, and samples were incubated at +50 °C for 1 h, followed by the addition of 8 μL propidium-iodide (1 mg/mL) and incubation at +37 °C for 2 h. Flow cytometric analysis of the cell cycle was carried out using BD FACSCanto™ II (BD Biosciences, San Jose, CA, USA). A total of 12,000 cells per sample were analyzed using a flow cytometer (BD Accuri C6). For the cell cycle phase measurements (including the sub-G1 phase), the sample size used for statistical analyses and figures was 4 (*n* = 4). Etoposide (1.25 µM) served as a positive control.

### 2.9. OTM Analysis

DNA damage was detected using the alkaline single-cell gel/comet assay. The assay detects DNA single-strand breaks, alkali labile sites, DNA–DNA/DNA–protein cross-linking, and single-strand breaks associated with incomplete excision repair sites. After cell harvest, some of the cell suspension (20 µL) was mixed with 75 µL of 0.5% low-melting agarose (VWR), and the mixture was placed on a microscope slide covered with 1% normal-melting agarose (VWR). The slides were kept on ice for 5 min, after which the cover slips were removed, and the cells were treated with a lysing solution (2.5 M NaCl, 100 mM Na_2_EDTA, 10 mM Tris, 1% Triton X-100, pH 10) for 1 h at +4 °C to liberate the DNA. After cell lysis, the slides were placed in a horizontal electrophoresis tank, and the DNA was allowed to unwind for 40 min in the alkaline electrophoresis buffer (1 mM EDTA and 300 mM NaOH, pH > 13). The electrophoresis was run for 20 min at 24 V/300 mA. Finally, the slides were neutralized with Tris buffer (0.4 M, pH 7.5) and fixed in ethanol (99% *v*/*v*). The nuclei were stained with ethidium bromide (100 cells per dose) and analyzed using the image analysis system (CASPLab v. 1.2.3b2). The Olive Tail Moment (OTM), calculated as ((tail mean − head mean) × tail% DNA/100), was the parameter used for statistical analysis. In the genotoxicity experiments, the sample size used for statistical analyses and figures ranged from 3 to 5 (*n* = 3–5), depending on PM sample. Benzo[a]pyrene (0.25 µM) served as a positive control in the genotoxicity analyses.

### 2.10. Statistical Analysis

Each cell experiment was conducted with a minimum of three separate replicates. The observed responses were evaluated against the control with regard to particle doses. The data were statistically analyzed in IBM SPSS Statistics 21 (SPSS Inc., Chicago, IL, USA). The results from the toxicological endpoints were evaluated by the non-parametric Mann–Whitney U test. Differences were considered to be statistically significant at *p* < 0.05.

### 2.11. Use of Generative AI

Generative AI model (Microsoft Copilot) was used to enhance the language in minor parts of this manuscript.

## 3. Results and Discussion

### 3.1. Fuels

Representative fuel samples were analyzed for PAH and Oxy-PAH compounds (Supplementary File S3). SD10 contained PAHs at a total concentration of 446 mg/L. RME, representing a generation of esterified biofuel, had a considerably lower PAH content (36 mg/L) than SD10. SME had the lowest PAH content (3 mg/L). In this study, HVO was chosen to represent a renewable diesel. HVO displayed a similar PAH concentration as RME. These results are in good agreement with the findings of others, where petroleum-based diesel fuels have been reported to contain the greatest amounts of PAH compounds compared to biodiesel and renewable diesel fuel [41,42].

### 3.2. Particle Size Distribution

In the present study, the substitution of SD10 with a 100% RME formulation resulted in a shift in the particle number size distribution towards the ultrafine range (<100 nm). We demonstrated a mono-modal size distribution with SD10, RME 5%, and both HVO fuels, with peak number diameters in the range of 80–100 nm (mobility diameter). Additionally, a bi-modal size distribution was observed when RME and SME fuels were used, with the second peak in the range of 30–50 nm (mobility diameter) (Supplementary File S4).

### 3.3. Chemical Composition of the Exhaust Particles

#### 3.3.1. Carbon Fractionation and Metals

**Figure 1:**
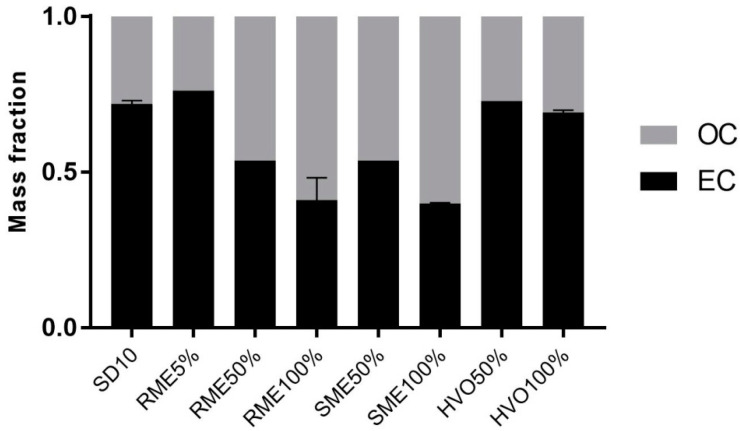
OC/EC mass fraction. Mass fraction of organic carbon (OC) and elemental carbon (EC) in PM emission particles from different biodiesels, renewable diesel, and SD10 diesel. Whiskers represent standard deviation. SD10 sample has *n* = 3; RME100%, SME100%, and HVO100% have *n* = 2; rest of the samples have *n* = 1.

The results from the carbon fractionation analysis (OC/EC) are presented in Figure 1. In general, carbonaceous matter dominated the PM. SD10, RME5%, and HVO samples had the highest mass fraction of EC, where it accounted for about two-thirds of EC. Correspondingly, RME50%, RME100%, SME50%, and SME100% had the highest amount of OC. Clearly, the use of biodiesel increased the amount of organic matter in the exhaust particles, resulting in about half of the total carbonaceous matter (TC). The results align well with previous studies indicating that higher biodiesel blends lead to increased organic matter emissions compared to conventional diesel and HVO [43,44].

When it comes to metal analysis, a comparison of the total elemental amounts of the blank filter with the results from the exhaust samples indicates that the majority of the analyzed elements were at trace levels (Supplementary File S5). Replicate samples from SD10 also indicated that values were close to the detection limit as they demonstrated high coefficients of variation for almost all elements. The only exceptions were zinc and copper, which showed both lower variability and detectable levels compared to the blank filter for all samples. Al, Fe, and Zn were the most abundant elements in the samples. Notably, fuels with high metal content are difficult to use in high blends in diesel engines when modern particle reduction after treatment technologies are used because the metals and other impurities may lead to a faster reduction of catalyst efficiency [45]. These results corroborate previous findings that carbonaceous components are abundant in biodiesel, renewable diesel, and diesel emissions [15,42,46].

#### 3.3.2. PAH and Oxy-PAH

DEP samples were analyzed for 44 PAHs and 12 Oxy-PAHs compounds, including those classified by the WHO as carcinogenic (Table 1 and Table 2). The total content of PAHs was highest in the RME5% and SD10 PM samples (223 ng/mg and 216 ng/mg, respectively) and lowest in the RME100% (58 ng/mg) and SME100% (64 ng/mg) particle samples. Among the 50% blend fuels, the highest PAH concentration (158 ng/mg) was demonstrated for HVO50%. Pyrene, fluoranthene, and phenanthrene were the most abundant PAHs in all the DEP samples. The genotoxic PAH concentrations in the samples followed a similar trend as the total PAH concentrations (see Table 1).

The proportion of heavy PAH compounds (molecular weight above 202 u) showed some variation as compared to total and genotoxic PAHs. PM from SD10 and RME5% contained the highest amounts of the heavy PAH compounds (30 and 31 ng/mg, respectively), followed by RME50% (24 ng/mg) and SME50% (25 ng/mg), as well as RME100% and SME100% samples (19 and 20 ng/mg, respectively). Surprisingly, HVO50% (18 ng/mg) and especially HVO100% (12 ng/mg) displayed the lowest concentrations of heavy PAHs (Table 1). Oxy-PAH levels in DEP samples roughly mirrored the pattern of the total PAH levels (Table 2).

In this study, the urban part of the ETC test protocol was used to generate the PM samples. The urban part simulates city driving with a maximum speed of 50 km/h and includes frequent starts, stops, and idling. The PAH emission of diesel engines is influenced by many factors, such as differences in engine technology, engine operating conditions (including testing protocols), and fuel properties (e.g., PAH and metal content).

Advancements in diesel engine technology have led to substantial reductions in PAH emissions. This is largely due to the use of the Diesel Particulate Filters (DPFs) and the effective burning of PAHs desorbing from the engine liner [47,48].

Engine load and speed also play a significant role in the formation and emission of PAHs. As the engine load increases, the combustion temperature and pressure rise, leading to a higher rate of PAH formation. This is attributed to the process of pyrosynthesis, which involves the transformation of lower molecular weight hydrocarbon compounds into larger PAHs [49]. The relationship between engine speed and PAH emissions is more complex. Initially, an increase in engine speed results in a sharp decrease in PAH emissions, likely due to more complete combustion that reduces the formation of PAHs. However, beyond a certain speed, PAH emissions may start to increase slightly. This could be attributed to less efficient combustion at very high speeds, leading to incomplete combustion and consequently, higher PAH emissions [50,51,52].

The properties of the fuel, such as carbon content, density, viscosity, sulfur content, and aromatic content, also significantly influence PAH emissions. For instance, an increase in carbon content or a reduction in sulfur content can lead to a respective rise or decrease in particulate emissions, which often contain PAHs. Furthermore, the type of fuel used, such as biodiesel compared to conventional diesel, can significantly influence PAH emissions. It is crucial to note that these effects can vary based on the specific design and operation conditions of the engine [53,54].

In general, the PAH profiles determined in this study resemble those previously reported by the present investigators and others [20,32,55]. Moreover, our results are consistent with the review by Bünger et al. [56], which indicates significantly lower levels of most light PAHs (molecular weight below 202 u) and total PAH in the exhaust PM when pure biodiesel or renewable diesel is used as compared to conventional diesel. PAH emissions from low biodiesel blends (5–20%) do not appear to differ significantly from those of conventional diesel [56]. This was also seen in the present study when RME5% was used.

### 3.4. Cytotoxicity

Cell viability was assessed with the (3-(4,5-dimethylthiazolyl-2)-2,5-diphenyltetrazolium bromide) test (MTT), and the results are shown in Supplementary File S6. None of the samples evoked a statistically significant cytotoxic response in this study. This indicates that cytotoxicity did not interfere with our other endpoints. In contrast to our findings, some studies have indicated that particle samples from conventional diesel, biodiesel, and renewable diesel can induce cell death at similar concentrations as those used in the current study. Jalava et al. [20] observed clear responses with the MTT assay using emission particles from pure RME and HVO fuel combustion, although PM from conventional diesel fuel produced an even greater effect. Jalava et al. [20] used the mouse macrophage cell line (RAW 264.7). Similarly, Martin et al. [57] reported cytotoxic effects caused by conventional diesel PM but not by biodiesel PM when BEAS-2B cells were used. The degree of cytotoxicity caused by biodiesel and renewable diesel emissions compared to conventional diesel fuels has yielded conflicting results. For example, it was observed that PM from RME100% caused a significantly higher release of lactate dehydrogenase (LDH), an enzyme indicative of necrotic cell death, compared to PM from conventional diesel fuel when RAW264.7 cells were used in the study [58]. Similar results as mentioned earlier have been reported in other studies employing human cell lines (3D in vitro model of the human epithelial airway barrier and BEAS-2), where RME PM emissions were found to be more toxic than PM from traditional diesel fuel combustion [59,60]. Variations in observed responses may be attributed to differences in the cell lines used or the engine characteristics.

**Figure 2:**
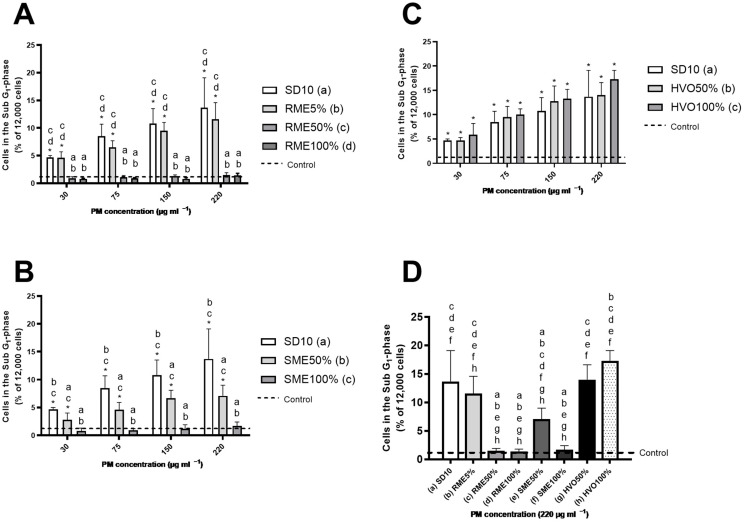
sub-G1. (**A**–**C**): A549 cells in sub-G1 phase after 24 h exposure to PM emission particles from different biodiesels, renewable diesel, and SD10 diesel. Bars represent four concentrations (30, 75, 150, and 220 mg mL^−1^); whiskers are the standard deviation (SD). The asterisks indicate statistical significance compared to the control (*p* < 0.05) analyzed using the nonparametric Mann–Whitney U test. The lowercase letters indicate a statistically significant response between samples (*p* < 0.05) based on a Mann–Whitney U test. (**A**) RME samples and SD10; (**B**) SME samples and SD10; (**C**) HVO samples and SD10. Graph (**D**): A459 cells in sub-G1 phase after 24 h exposure to PM emission particles from different biodiesels and SD10 diesel. Bars represent concentrations of 220 mg mL^−1^; whiskers are the SD. The lowercase letters show a statistically significant difference between samples (*p* < 0.05) as determined by a Kruskal–Wallis test.

In the present study, apoptotic cell death was analyzed by a flow cytometric assay of PI-stained fixed cells [61] (Figure 2), where SD10, RME5%, SME50%, and both HVO PM samples evoked a dose-dependent, statistically significant increase in the sub-G1 response compared to the control. RME50%, RME100%, and SME100% did not cause any sub-G1 response. The SME50% sample induced a response that was approximately half that of the SD10 sample. When comparing the highest PM dose of each diesel sample (Figure 2D), HVO100% elicited the strongest response in the sub-G1 analysis, followed by HVO50%, SD10, and RME5%. Interestingly, RME50%, RME100%, and SME100% did not exhibit a sub-G1 effect even at the highest PM dose. Full cell cycle analysis data are shown in Supplementary File S7.

The overall percentage of apoptotic cells did not exceed 20% even at the highest PM dose, indicating that the acute cytotoxic potential of the DEPs used in this study was low, a finding that is corroborated by our MTT results and is consistent with other reports [20,62]. Moreover, similar levels of apoptosis induction by DEPs from conventional diesel fuel have been reported in other studies, comparable to our findings when using HaCaT and A549 cells [63]. However, in the previously mentioned study, the detected apoptosis remained at control levels when a biodiesel blend (FAME20%) was used [63]. Interestingly, Jalava et al. [20,64] found no differences in the sub-G1 responses when assessing diesel particle samples in a mouse macrophage cell line (RAW 264.7) exposed to emissions from conventional diesel, RME100%, and HVO100%, at similar concentrations as used in the current study. These results confirm that the engine type and running conditions can modify the chemical composition of the emitted PM, potentially affecting the mechanisms through which DEPs induce cell death. For example, Jalava et al. [20,64] used the C1 cycle and the Braunschweig cycle in their studies. The running conditions in the present study were the ETC, urban part, as we aimed to study a situation mimicking typical urban transportation. Differences in the detected responses may also be attributed to the use of various cell lines, each of which responds differently to PM exposure.

### 3.5. ROS Production

**Figure 3:**
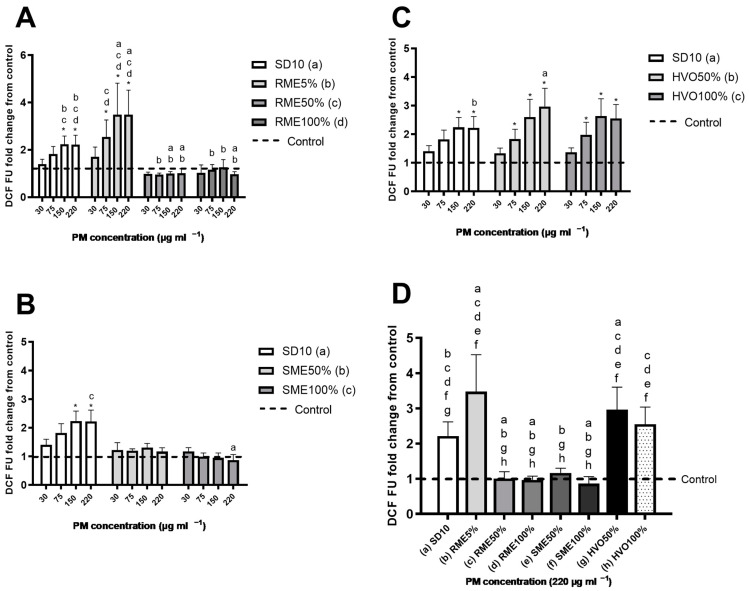
Detected ROS response. (**A**–**C**): Using a DCF assay, the ROS response in A549 cells was measured following 24 h exposure to particulate matter emissions from diverse biodiesels, renewable diesel, and SD10 diesel. Bars represent four concentrations (30, 75, 150, and 220 mg mL^−1^); whiskers are the standard deviation (SD). The asterisks indicate statistical significance compared to the control (*p* < 0.05) analyzed using the nonparametric Mann–Whitney U test. The lowercase letters indicate a statistically significant response between samples (*p* < 0.05) based on Mann–Whitney U test. (**A**) RME samples and SD10; (**B**) SME samples and SD10; (**C**) HVO samples and SD10. Graph (**D**): ROS response in A459 cells after 24 h exposure to PM emission particles from different biodiesels, renewable diesel, and SD10 diesel. Bars represent concentrations of 220 mg mL^−1^; whiskers are the SD. The lowercase letters show a statistically significant difference between samples (*p* < 0.05) as determined by Mann–Whitney U test.

Figure 3 shows the ROS production in A549 cells following exposure to various diesel emission PM samples. All samples, except those from RME50%, RME100%, and SME100%, significantly increased ROS production when responses were compared to the corresponding control. The PM samples from the RME5% and HVO50% were the most potent in causing oxidative stress in A549 cells on an equal mass basis, followed by the SD10 and HVO100% PM samples (Figure 3D). Interestingly, the measured ROS levels generally aligned with the detected sub-G1 results except for the SME50% PM sample. It is worth noting that SME50% also induced the lowest sub-G1 response among samples with a statistically significant response (Figure 2). The samples that triggered the strongest ROS response also contained the highest amounts of EC, which constituted more than half of their total PM mass.

When comparing our results to the literature, similar results were reported by Jalava et al. [64], who found that particles from pure conventional diesel and renewable diesel (HVO100%) induced a concentration-dependent ROS generation in a mouse macrophage cell line (RAW 264.7). Moreover, HVO100% particles induced significantly more ROS than pure diesel particles in that study. In contrast, a study by Libalova et al. [65] reported no significant increase in ROS generation in BEAS-2B cells by conventional diesel, HVO100%, RME100%, and RME30% particles. Similarly, Lankoff et al. [62] did not observe any differences in the ROS effects on BEAS-2B and A549 cells when comparing PM derived from FAME7%, FAME20%, and 2nd generation biodiesel fuel containing 13 vol.% synthetic HVO and 7 vol.% FAMEs. Discrepancies between these results may arise not only from different engines, running modes, and fuel types, but also from differences in the experimental setups used to assess the intracellular ROS generation. However, it has been suggested that EC in PM correlates with detected cellular ROS effects [66]. Indeed, using an abiotic DTT assay, a strong positive correlation between high oxidative potential (OP) and EC has been detected from the engine-derived PM [67]. Similarly, oxygenated PAHs have been noted to be important contributors to the OP of the ambient particles and are considered to be more toxic than their parent PAHs [68]. Notably, in the current study, SD10, RME5, and HVO50 had the highest Oxy-PAH concentrations among the samples.

### 3.6. Genotoxicity

**Figure 4:**
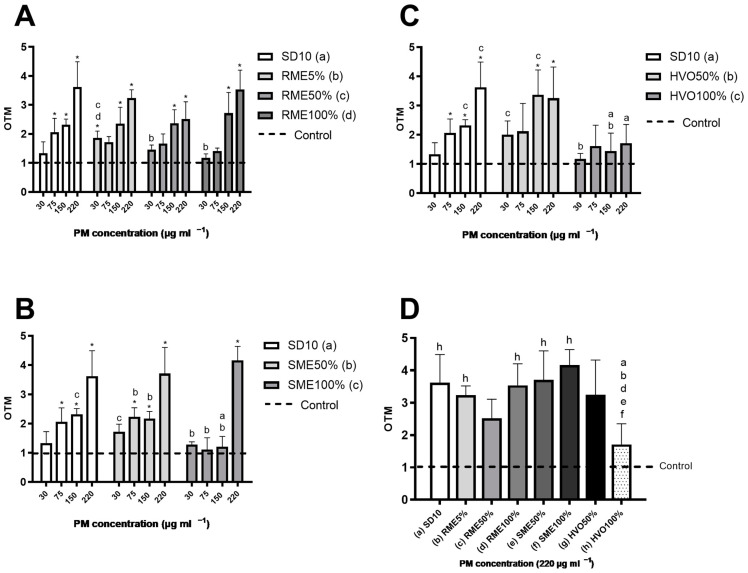
OTM. (**A**–**C**): Using a comet assay, the genotoxic response in A549 cells was measured following 24 h exposure to PM emissions from diverse biodiesels, renewable diesel, and SD10 diesel. Bars represent four concentrations (30, 75, 150, and 220 mg mL^−1^); whiskers are the standard deviation (SD). The asterisks indicate statistical significance compared to the control (*p* < 0.05), analyzed using the nonparametric Mann–Whitney U test. The lowercase letters indicate a statistically significant response between samples (*p* < 0.05) based on a Mann–Whitney U test. (**A**) RME samples and SD10; (**B**) SME samples and SD10; (**C**) HVO samples and SD10. Graph (**D**): Genotoxic response in A459 cells after 24 h exposure to PM emission particles from different biodiesels, renewable diesel, and SD10 diesel. Bars represent concentrations of 220 mg mL^−1^; whiskers are the SD. The lowercase letters show a statistically significant difference between samples (*p* < 0.05) as determined by Mann–Whitney U test.

All the samples, except for HVO100%, showed an increase in the olive tail moment (OTM) value, which indicates DNA fragmentation in cells (Figure 4). The SD10 and RME samples demonstrated a clear dose-dependent response in A549 cells when OTM was measured. In contrast, the SME100% PM samples did not show this pattern and only caused a significant increase in OTM at the highest concentration. HVO100% exhibited the lowest OTM value. Indeed, when the highest dose of PM samples was compared, they were quite similar to each other (Figure 4D), except for the HVO100% PM sample. Interestingly, despite RME having the lowest levels of PAHs and also the lowest apoptotic and ROS responses, it showed a significant increase in OTM, similar to SD10. On the other hand, HVO100% had a much lower OTM but exhibited greater responses in the sub-G1 and ROS analyses. These observations suggest that the genotoxic response, as indicated by OTM, may be driven more by the presence of heavy PAHs (e.g., benzo[a]pyrene) rather than the overall quantity of PAHs. In many previous studies, authors have argued that the amount of PAH compounds in a sample correlates well with the elicited OTM response [20,64,69]. In summary, it appears that the OTM results are influenced more by the quantity and quality of PAHs, particularly the effects induced by heavy PAH compounds, rather than the type of fuel used or the engine type.

### 3.7. Inflammation

The studied DEP samples induced only a low inflammatory response as determined by the pro-inflammatory cytokine release (Supplementary File S8). SD10 and RME5% induced the highest IL-6 response, reaching approximately 125 mL^−1^ (the control level was approximately 100 mL^−1^). The RME100% sample significantly reduced IL-6 levels compared to the control. Other samples did not induce any response when they were compared to control. A highly similar result was seen when IL-8 was measured from cell culture medium. Indeed, only one dose of SD10 caused a statistically significant increase in IL-8 release when responses were compared to the control. Similar to the IL-6 results, RME100% caused a statistically significant decrease in IL-8 release compared to control.

Our results are in good agreement with other studies. In animal studies, conventional diesel-, biodiesel-, and renewable diesel-derived PM were shown to cause low to moderate inflammation in mice and rats [46,70,71]. Moreover, many in vitro studies using various cell lines have not found any differences in potency to induce inflammation reactions between conventional diesel, renewable diesel, and biodiesel PM [20,72]. However, some studies have reported that PM from biodiesel fuel was more inflammatory than PM from conventional diesel, inducing a higher IL-6 release in a human cell line (BEAS-2B) [73]. Similarly, SME100% induced greater IL-6 responses in BEAS-2B cells compared to petroleum diesel [74]. Additionally, FAME20% fuel blends induced greater cytokine responses in two used human cell lines (BEAS-2B and THP-1) compared to conventional diesel [75]. To summarize, the data are inconclusive as to whether PM from biodiesel or renewable diesel is equally or more potent at causing inflammation compared to conventional diesel fuel PM [18].

### 3.8. Methodological Considerations

A heavy-duty diesel engine was used to generate exhaust particles from various fuels using a single well-characterized running condition, which had been previously used in many human exposure chamber studies with petrodiesel and biodiesel [24]. However, the use of this setup limits the generalizability of the findings to other engines, operating conditions, and fuel blends. Different engines may produce varying emissions and thus different toxicological responses.

Although the used PM collection method is efficient, it may not capture the full spectrum of particle sizes and chemistry in diesel exhaust. However, the current method has been proven to be at least as good as the Dekati^®^ Gravimetric Impactor collection method [28]. Moreover, the size of the collected particles can change during collection in water. Additionally, the handling of PM samples, such as thawing and freezing, can lead to the formation of aggregates or other alternative PM surface structures.

We conducted a comprehensive chemical analysis of this study’s PM samples. However, the sample sizes for many chemical analyses are small (e.g., *n* = 1 for some fuel blends). This small sample size can affect the reliability of the chemical analysis results.

The use of A549 cells provides valuable insights into the cellular responses to PM but does not replicate the complexity of human (or animal) respiratory systems. This limitation affects the comparability of these results to animal and human studies. Moreover, the exposure method, where PM is pipetted into the cell culture medium and cells are at the bottom of the well, is not optimal and does not mimic real-life exposure situations very well. However, this method is fast and cost-efficient, making it suitable for PM screening studies. Finally, in this study, the cells were exposed to particulate matter for 24 h, which does not accurately reflect real-world exposure scenarios where individuals are exposed to much lower concentrations over longer periods. This limitation affects the generalization of the current toxicological findings, for example, in human exposure scenarios.

## 4. Summary

In the present study, we compared the toxicity of DEPs from the combustion of conventional petroleum diesel, renewable diesel, and biodiesel fuels in relation to their chemical properties. The total content of PAHs was highest in DEPs from 100% petroleum diesel (SD10) and RME5% biodiesel (in 95% SD10). This was followed by HVO50% and SME50% (in 50% SD10). The analysis of OC and EC mass fractions showed that EC dominated the SD10, RME5%, HVO50%, and HVO100% DEPs.

HVO100% triggered the strongest sub-G1 apoptotic response, followed by HVO50%, SD10, and RME5% n 95% SD10. In contrast, RME50%, RME100%, and SME100% caused no sub-G1 effect. The reactive oxygen species (ROS) responses were generally aligned with the sub-G1 results, suggesting a relationship between oxidative stress and the observed cytotoxicity.

When genotoxicity was assessed using the comet assay, there were only moderate differences between the DEP samples. The detected genotoxicity did not appear to be directly connected to apoptotic or ROS responses. Instead, genotoxicity was associated with heavy PAH levels rather than the total DEPs PAH content.

These findings highlight the complexity of the toxicological effects of diesel emissions and underscore the need for further investigation into the specific chemical constituents that drive these adverse health effects. Future studies should focus on elucidating the mechanisms by which different PAH fractions and carbon components contribute to toxicity and exploring potential mitigation strategies to minimize the health risks associated with diesel emissions.

## 5. Conclusions

This study demonstrates that PM emissions from both the reference standard petroleum diesel (SD10) and alternative diesel fuels, including RME, SME, and HVO, exhibit significant toxic effects on human alveolar epithelial cells in vitro. The results suggest that the apoptotic and ROS responses are primarily associated with EC and Oxy-PAH content. Genotoxic responses appeared to be related to the DEPs’ heavy PAH content. These findings are coherent with our recent studies of controlled chamber biodiesel exposure in human subjects exploring cardio-pulmonary endpoints.

## Figures and Tables

**Table 1:** PAH content from different DEPs (ng/mg PM mass).

Fuel	SD10		RME5%	RME50%	RME100%		SME50%	SME100%		HVO50%	HVO100%	
*n*	3	R	1	1	2	R	1	2	R	1	2	R
PAHs												
fluorene	1.3	6	1.5	1.3	1	16	1.6	1.2	5	1.5	1.4	41
phenanthrene	41	7	47	17.6	7.5	9	33	13	5	39	26	6
**anthracene**	8.4	12	9.1	3.3	1.4	7	7.1	2.4	10	7.4	4	5
3-Methylphenanthrene	13	15	12	5.3	2.5	9	6.2	3.8	3	9.2	5.8	6
2-Methylphenanthrene	16	15	14	7.5	3.8	9	8.2	5	5	11	8.1	5
2-Methylanthracene	5.2	13	5	2.1	0.5	52	0.3	0.2	47	1.2	2.4	9
9-Methylphenanthrene	20	14	17	7.6	2.7	36	7	2.1	1	12	8.3	9
**1-methylphenanthrene**	16	14	16	8.6	3.4	30	9	2.4	1	12	8.4	7
1,7-Dimethylphenanthrene	8.4	10	11	5.6	2.6	31	4.8	2.6	10	6.6	4	6
Σ Dimethyl-178 ^a^	77	13	66	33	13	28	33	15	2	48	31	5
**fluoranthene**	27	0.2	30	20	7.8	25	16	6.4	3	20	13	1
pyrene	31	1	31	22	8.6	31	19	6.7	0.2	21	14	2
*3-Methylfluoroanthene* ^b^	5.5	1	6.0	4.5	2.8	17	4.4	3.1	3	3.5	2.3	2
*Retene*	5.3	8	5.5	3	1.4	37	2.7	1.3	2	3.2	2.2	4
*4-Methylpyrene*	4.5	1	4.3	2.8	1.5	26	2.9	1.7	9	2.7	1.4	1
*1-Methylpyrene*	2.2	4	2.3	1.8	1	31	1.8	1.1	5	1.4	0.6	3
*benz(a)anthracene*	1.2	6	1.1	1.4	1	20	1.3	1.2	0.5	0.6	0.4	9
*triphenylene + chrysene*	3.9	4	3.8	4	3.6	8	4.1	3.8	2	2.4	1.6	4
*3-Methylchrysene* ^b^	0.8	8	0.8	0.8	0.6	5	0.8	0.7	3	0.5	0.4	11
*6-Methylchrysene*	0.3	10	0.3	0.4	0.3	1	0.3	0.4	9	0.2	0.1	7
*Σ Methyl-228* ^c^	2	7	2	2.2	2.1	1	2	2.2	1	1.3	1.1	6
*benzo(b + j)fluoranthene*	1.3	3	1.3	1.3	1.5	21	1.4	1.6	1	0.7	0.4	1
*benzo(k)fluoranthene*	0.3	25	0.2	0.2	0.3	8	0.4	0.3	16	0.1	0.1	1
*benzo(e)pyrene*	1.2	7	1.3	1.2	1.3	18	1.3	1.5	6	0.6	0.5	4
*benzo(a)pyrene*	0.5	9	0.4	0.5	0.5	29	0.3	0.5	16	0.2	0.1	2
*perylene*	0.05	19	0.06	0.03	0.04	2	0.06	0.1	10	0.03	0.01	17
*indeno(1,2,3-cd)pyrene*	0.5	13	0.6	0.3	0.4	12	0.4	0.4	1	0.4	0.4	48
*dibenz(a, h + a, c)anthracene*	0.1	28	0.1	0.1	0.1	5	0.1	0.1	9	0.05	0.1	24
*benzo(ghi)perylene*	0.7	16	0.9	0.4	0.7	12	0.5	0.5	12	0.2	0.2	5
Sum of 44 PAHs	216	7	223	124	58	18	135	64	0.3	158	107	5
**Sum of genotoxic PAHs**	61	5	66	42	22	19	42	21	1	48	30	4
*Sum of heavy PAHs*	30	3	31	25	19	15	25	20	0.5	18	12	4

Genotoxic PAHs (WHO, 1998) are presented in bold. Heavy PAHs are presented in italics. R stands for relative standard deviation in percent. ^a^ Sum of 7 PAH isomers with a molecular mass of 206 u quantified using the response factor for 1,7-Dimethylphenanthrene. ^b^ Including one additional not identified isomer. ^c^ Sum of 6 PAH isomers with a molecular mass of 242 u quantified using the response factor for 6-Methylchrysene.

**Table 2:** Oxy-PAHs (ng/mg PM mass).

Fuels	SD10		RME5%	RME50%	RME100%		SME50%	SME100%		HVO50%	HVO100%	
*n*	3	R	1	1	2	R	1	2	R	1	2	R
*Ketones*												
9H-fluoren-9-one	19	8	22	7.5	4.2	15	18	7.6	7	23	16	4
1H-phenalene-one	59	1	43	26	16	25	30	16	4	40	31	7
10H-anthracene-9-one	3	20	1.7	1.2	<0.4		na	na		0.6	1.2	9
4H-cyclopenta[def]phenanthren-4-one	10	6	12	9.7	7.4	4	12	10	4	12	8.2	4
11H-benzo[a]fluorene-11-one	4.4	9	3.6	4.6	4.1	9	4.6	5.4	3	3.4	2.2	8
7H-benzo[de]anthracene-7-one	4.5	5	3.7	4.0	4.2	10	4.4	5.0	9	3.4	1.9	13
6H-benzo[cd]pyrene-6-one	1.7	11	1.7	1.5	1.9	30	1.6	2.0	2	1.0	0.8	10
*Quinones*												
anthracene-9,10-dione	33	3	28	14	6.8	30	17	5.1	1	22	20	5
2-methyl-anthracene-9,10-dione	9.3	6	7.0	4.1	na		na	na		2.6	3.6	1
2-ethyl-anthracene-9,10-dione	0.5	18	0.4	0.4	0.5	9	0.3	0.4	14	0.3	0.4	2
benz[a]anthracene-7,12-dione	1.2	15	0.8	1.3	1.0	23	0.9	1.1	8	0.8	0.7	35
naphthacene-5,12-dione	0.3	20	0.2	0.3	0.2	28	0.1	0.5	10	0.2	0.1	23
Sum of 12 Oxy-PAHs	146	2	124	74	46	16	89	53	3	110	86	6

na: not available due to sample matrix interference. R stands for relative standard deviation in percent.

## Data Availability

The relevant datasets supporting the conclusions of this article are included within the article, and all datasets used and analyzed during the current study are available from the corresponding author upon reasonable request.

## Supplementary Materials

The following supporting information can be downloaded at https://www.mdpi.com/article/10.3390/jox14040080/s1: Supplementary File S1: Particle collection; Supplementary File S2: Chemical analysis; Supplementary File S3: Fuel PAH; Supplementary File S4: DMPS; Supplementary File S5: Metals; Supplementary File S6: MTT; Supplementary File S7: Cell cycle; Supplementary File S8: Inflammation.
