# Supplementary material for: The Toxic Effects of Petroleum Diesel, Biodiesel, and Renewable Diesel Exhaust Particles on Human Alveolar Epithelial Cells
Source: J Xenobiot. 2024 Oct 9;14(4):1432–49. doi: 10.3390/jox14040080 (PMC11503417; doi:10.3390/jox14040080)
Supplement: Supplementary file 1 [file jox-14-00080-s001.zip › Supplementary file S1_Particle collection.pdf]

Supplementary file S1.

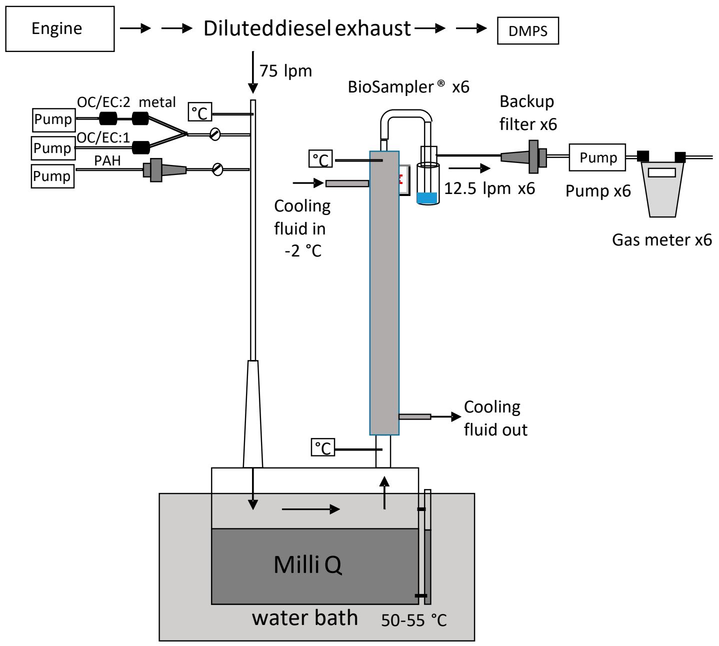

Figure S1. Schematic overview of the system for collection of diesel exhaust particles

Diesel Particle collection for chemical analysis and determination of mass concentration

Figure S1 gives a schematic overview of the system for collection of DEPs. During each BioSampler collection period of 7-8 h, a minimum of three sets of filter samples, corresponding to the integers of the ETC cycle time (urban part, n x 10 minutes), were collected. For gravimetric particle mass determination and PAH/Oxy-PAH analysis, DEPs were sampled at a flow of 7.0 l/min on pre-weighed PTFE filters (Zeflour^{TM}, 47 mm, 2.0 µm, Pall Corp., NY, USA) mounted on an in-line metal filter holder using a pump (Leland Legacy, SKC Inc). For OC/EC determination, a two-port system was used according to Turpin et al. [1]; therefore, the stream of diluted exhaust was split into two halves. The first half was directed through OC/EC:1 (Quartz-filter, Pallflex Tissuequartz, 47 mm, Pall Corp., NY, USA) using a pump set at 4.0 l/min (GilAir Plus, Sensidyne, USA), and the second half through two filters in series: a PTFE filter followed by OC/EC:2 (Quartz-filter, backup for OC/EC) with a pump set at 4.0 l/min (GilAir Plus, Sensidyne, USA). Filters in this two-port system were mounted on conductive filter cassettes (SKC inc., PA, USA), and the PTFE filter was used for metal analyses. All flows were checked with a primary flow meter (Defender 520, Mesa Labs, LA, USA).

Measurement of particle number size distribution

In a subsequent dilution step (see Figure S1), yielding roughly a PM mass concentration of 300 µg/m^3, a differential mobility particle sizer (DMPS), consisting of an electrostatic classifier (model 3071, TSI Inc. Shoreview, MN, USA) and a condensation particle counter (CPC, model 3010, TSI Inc.), was used to measure particle number size distribution of the sub-micrometer (<1 µm) fraction. Air flows were monitored with mass flow meters (model 4199, TSI Inc.), and sheath, sample, and CPC flows were kept at 3.0 l/min, 0.3 l/min, and 1.0 l/min, respectively, using needle valves and external pumps. The working fluid in the CPC was 1-butanol.

Diesel Particle Collection for in vitro testing

A custom-designed collection system based on saturation/condensational growth together with Bioaerosol impingers (BioSampler®, SKC inc, Eighty-Four, PA, USA) was employed to collect particles for in vitro

testing. A sampling system of this type has been shown to allow efficient collection of both ultrafine and fine particles, and it preserves a high level of physical and chemical characteristics of sampled aerosols when concentrated into a biocompatible liquid medium. Diluted exhaust gas with a mean temperature of 40 °C was drawn through the humid headspace above the surface of ultrapure water (Milli-Q® Advantage A10, Merck Millipore, Germany) maintained at 55±2 °C to saturate the gas stream. The water level was controlled regularly by inspecting an auxiliary column and typically refilled two times per sampling period. The particle–vapor mixture then entered a condensation tube connected to a circulating chiller (50:50 ethylene glycol:H_2O, LTC 20-40, Grant Instruments Ltd., UK) maintained at -2 °C to promote condensational particle growth. The dimensions of the saturator chamber and the condensation tube resulted in residence times of 3.4 s and 0.34 s, respectively, which are close to residence times obtained in similar constructions [2-4] . Furthermore, a good compliance was also obtained for the gas temperature decrease (10±1 °C) through the condensation tube. From the top of the condensation tube, the exhaust stream was directed through six BioSamplers® via a six-port manifold. Downstream of each BioSampler®, a backup filter (PTFE) was mounted on an in-line metal holder. The individual BioSampler® flows (12.5 l/min/sampler) provided by pumps (Model DOA-P109-FD, Gast Manufacturing, Inc., MI, USA) were regularly checked with a primary flow meter (Defender 520, Mesa Labs, LA, USA), and backup filters were replaced when a 10 % decrease in the flow was observed. The total air volume sampled through each BioSampler® was measured by gas meters placed downstream of the pumps. Temperatures were monitored in the incoming diluted exhaust and up- and downstream of the condensation tube (see Figure S1).

The six BioSampler® particle suspensions from each sampling period were combined (~120 ml) and stored at 4 °C until further processing. The corresponding backup filters were individually probe sonicated (Vibracell™, Sonics & materials inc., CT, USA) until the filter was thoroughly stripped of particles, typically three times for 1 minute in a beaker using 3x5 ml MeOH. The extraction solvents were combined and concentrated to ~1 ml in a rotary evaporator (Rotavapor R-114, BÜCHI Labortechnik AG, Switzerland) before addition of 2 ml MilliQ water and subsequent concentration to ~2 ml. This concentrated backup filter portion was then combined with the main Biosampler® suspension. The mean DEP concentration in final suspensions from all sampling periods was 2.6±0.6 mg/ml.

Reference

1. Turpin, B.J.; Saxena, P.; Andrews, A. Measuring and simulating particulate organics in the atmosphere: Problems and prospects. *Atmos. Environ.* **2000**, *34*, 2983–3013.
2. Geller, M.D.; Biswas, S.; Fine, P.M.; Sioutas, C. A new compact aerosol concentrator for use in conjunction with low flow-rate continuous aerosol instrumentation. *J. Aerosol Sci.* **2005**, *36*, 1006–1022.
3. Kim, S.; Jaques, P.A.; Chang, M.; Froines, J.R.; Sioutas, C. Versatile aerosol concentration enrichment system (VACES) for simultaneous in vivo and in vitro evaluation of toxic effects of ultrafine, fine and coarse ambient particles Part I: Development and laboratory characterization. *J. Aerosol Sci.* **2001**, *32*, 1281–1297.
4. Sioutas, C.; Kim, S.; Chang, M. Development and evaluation of a prototype ultrafine particle concentrator. *J. Aerosol Sci.* **1999**, *30*, 1001–1017.
