# Supplementary material for: The Toxic Effects of Petroleum Diesel, Biodiesel, and Renewable Diesel Exhaust Particles on Human Alveolar Epithelial Cells
Source: J Xenobiot. 2024 Oct 9;14(4):1432–49. doi: 10.3390/jox14040080 (PMC11503417; doi:10.3390/jox14040080)
Supplement: Supplementary file 1 [file jox-14-00080-s001.zip › Supplementary file S2_Chemical analysis.pdf]

Supplementary file S2.

**Chemical analysis**

Particle concentration

Particle mass concentration in the diluted exhaust flow was determined by the DEP mass collected on the polytetrafluoroethylene (PTFE) filters. After overnight stabilization, each filter was weighed until two readings differed by less than 0.02 mg within a period of 3 h. A number of laboratory filter blanks were also treated and weighed in the same way to verify the accuracy and consistency of the measurements. Variation in particle mass concentration during each 7-8 h sampling period was typically below 10 % RSD, as determined from three to five filters sampled evenly distributed over each period.

Metals

The PTFE filters were subjected to a microwave-assisted pressurized acid digestion procedure and analyzed for metals by inductively coupled plasma mass spectrometry (ICP-MS).

Carbon fractionation analysis

The analysis of particulate carbon fractions was performed by a standard thermal-optical method to determine the content of organic carbon (OC) and elemental carbon (EC). Standard 47mm tissue quartz filters and 47mm PTFE membrane filters were used according to a well-established sampling methodology [1], with subsequent carbon analysis following the EUSAAR 2 thermal protocol [2]. The EUSAAR 2 protocol is defined by four steps in a helium atmosphere, followed by four steps in a helium/oxygen atmosphere, each step with different temperatures and residence times.

PAHs and Oxy-PAHs

PTFE filters for PAH/Oxy-PAH analysis were extracted as follows: Three filters, which were sampled evenly distributed over the 7-8 h sampling period, were jointly subject to ultrasonic extraction with 3 x 5 ml dichloromethane (DCM) for 10 min each. Before extraction, internal standard was added (90 ng each of 1aphthalene-$d_8$, acenaphtene-$d_{10}$, phenanthrene-$d_{10}$, anthracene-9.10-dione-$d_8$, chrysene-$d_{10}$, perylene-$d_{10}$, and benzo(ghi)perylene-$d_{12}$). The combined extract was filtered, and after a solvent change to hexane, the extract was cleaned up and fractionated on a silica column, resulting in a DCM eluate containing PAHs and Oxy-PAHs. After evaporation, the solvent was changed to toluene, and 100 ng recovery standard (fluoranthene-$d_{10}$) was added. This extract was analyzed for PAHs and Oxy-PAHs by GC-MS operated in selected ion monitoring (SIM) acquisition mode, using at least two ions (m/z values) for each analyte. For increased sensitivity compared to the previous protocol, the volume of the final extract was reduced to 50 µl, GC injection volume was increased to 2 µl, MS electron multiplier gain factor was increased to 4, and the MS temperatures of the ion source and quadrupole were increased to 300 °C and 180 °C, respectively.

Joint calibration solutions of authentic standards were prepared in toluene from PAH standard mix SRM 1491 and 1491a (NIST, Gaithersburg, MD, USA) and from individual Oxy-PAH standards (Sigma-Aldrich, MO, USA, and Chiron AS, Norway). GC-MS analysis results from these calibration solutions, prepared in five concentrations and spiked with internal and recovery standards, were used for the identification and quantification of 44 PAHs and 12 Oxy-PAHs.

QA/QC

For positive identification, the following detection criteria were used: a signal-to-noise (S/N) ratio of 3:1, retention time within ±2 s, and isotope ratios for the two most abundant ions within 20% compared to authentic standards. All calibration standards were analyzed both before and after analysis of particle extracts. Additionally, one selected calibration standard was analyzed after every fifth particle extract. Calibration curves showed good linearity (average $r^2 = 0.999$) for all reported analytes. A number of solvent blanks, extracted method blanks, and field blanks were analyzed as well. Additionally, two replicates (~5 mg each) of standard reference material 1650b (Diesel Particulate Matter,

NIST, Gaithersburg, MD, USA.) were extracted and analyzed to check the accuracy and reproducibility of the method when applied for DEPs (Table S1).

**Table S1. Analyzed and certified/reference PAH mass fraction values (µg/g) for standard reference material SRM 1650b**

| | Cert.Conc. | SRM1650b_1 | SRM1650b_2 |
|---|---|---|---|
| **phenanthrene** | 66 | 56 | 55 |
| **3-Methylphenanthrene** | 57 | 49 | 48 |
| **2-Methylphenanthrene** | 72 | 63 | 61 |
| **9-Methylphenanthrene** | 37 | 33 | 32 |
| **1-methylphenanthrene** | 32 | 29 | 29 |
| **fluoranthene** | 48 | 47 | 44 |
| **pyrene** | 44 | 42 | 39 |
| **benz(a)anthracene** | 6.5 | 5.4 | 5.3 |
| **triphenylene+chrysene** | 23 | 24 | 23 |
| **benzo(b+j)fluoranthene** | 10 | 12 | 12 |
| **benzo(k)fluoranthene** | 2.3 | 2.8 | 2.7 |
| **benzo(b+k+j)fluoranthene** | 12 | 15 | 14 |
| **benzo(e)pyrene** | 6.4 | 8.0 | 7.6 |
| **benzo(a)pyrene** | 1.3 | 1.1 | 1.0 |
| **perylene** | 0.17 | 0.15 | 0.15 |
| **indeno(1.2.3-cd)pyrene** | 4.5 | 4.7 | 4.8 |
| **dibenz(a.h+a.c)anthracene** | 0.80 | 1.1 | 1.0 |
| **benzo(ghi)perylene** | 6.0 | 6.1 | 5.8 |
| | **Ref. Conc.** | **SRM1650b_1** | **SRM1650b_2** |
| **fluorene** | 0.76 / 1.3 | 0.99 | 0.86 |
| **anthracene** | 1.6 / 7.6 | 3.1 | 3.1 |
| **2-Methylanthracene** | 0.6 / 5.9 | 3.8 | 4.3 |
| **1.7-Dimethylphenanthrene** | 17 | 16 | 16 |
| **4H-cyclopenta[def]phenanthren-4-one** | 15.6 | 15 | 14 |
| **1+3-Methylfluoroanthene*** | 9.3 | 12 | 11 |
| **4-Methylpyrene** | 5.13 | 5.9 | 5.7 |
| **1-Methylpyrene** | 2.1 | 1.7 | 1.6 |
| **6-Methylchrysene** | 1.6 | 1.5 | 1.4 |

*Analyzed samples include one additional non-identified isomer

## Reference


1. Turpin, B. J.; Saxena, P.; Andrews, E. Measuring and simulating particulate organics in the atmosphere: problems and prospects. Atmos. Environ. 2000, 34 (18), 2983−3013.
2. Cavalli, F.; Viana, M.; Yttri, K. E.; Genberg, J.; Putaud, J. P. Toward a standardised thermal-optical protocol for measuring atmospheric organic and elemental carbon: the EUSAAR protocol. Atmos. Meas. Tech. 2010, 3 (1), 79−89.
