# Supplementary material for: The Toxic Effects of Petroleum Diesel, Biodiesel, and Renewable Diesel Exhaust Particles on Human Alveolar Epithelial Cells
Source: J Xenobiot. 2024 Oct 9;14(4):1432–49. doi: 10.3390/jox14040080 (PMC11503417; doi:10.3390/jox14040080)
Supplement: Supplementary file 1 [file jox-14-00080-s001.zip › Supplementary file S6_MTT.pdf]

Supplementary file S6. MTT results.

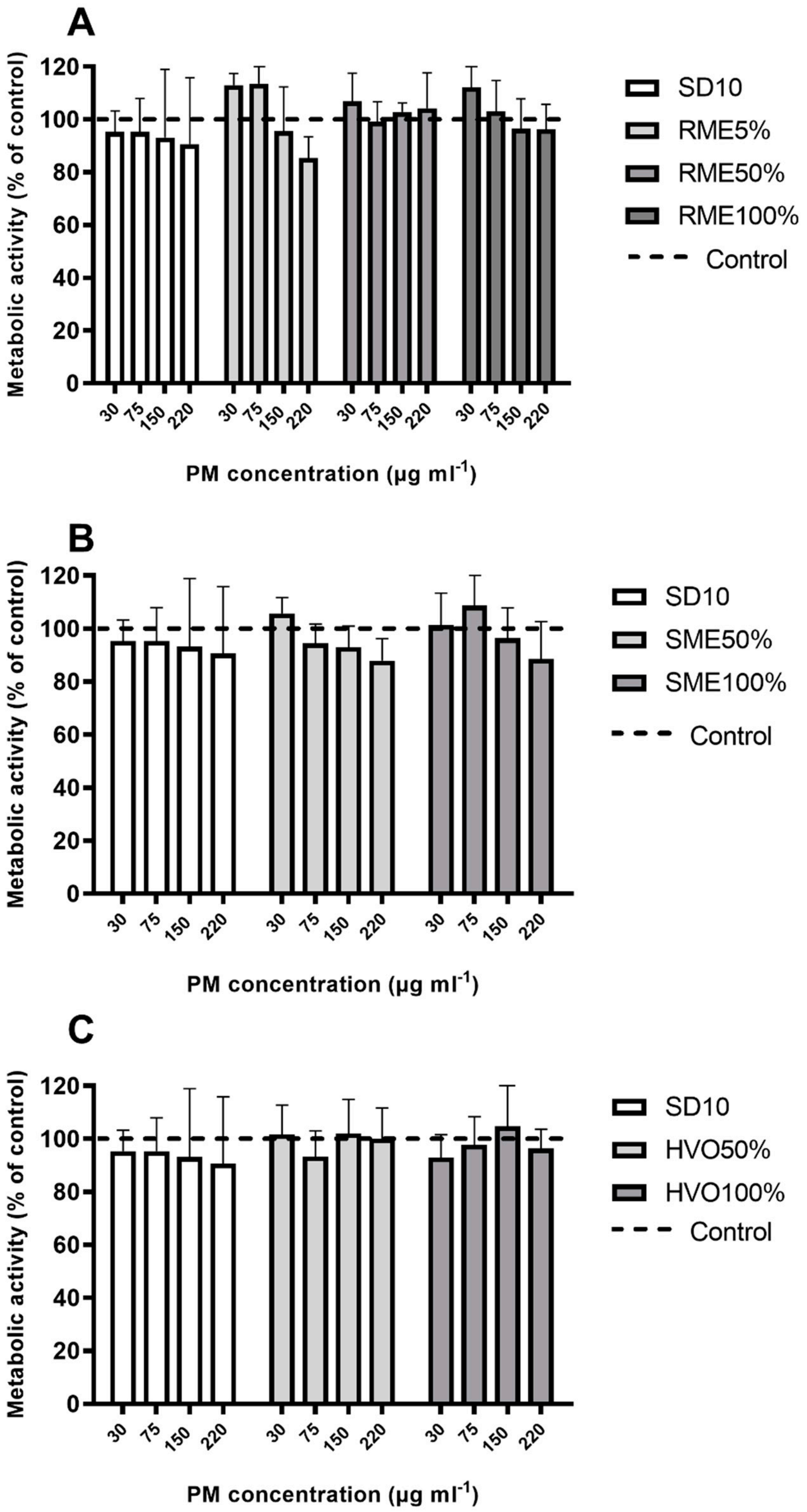

Cellular metabolic activity assessed with the MTT test after 24 h exposure to PM emission samples from different biodiesels, renewable diesel, and SD10 diesel. Bars represent four concentrations (30, 75, 150, and 220 mg ml^{-1}); whiskers are the standard deviation (SD). A) RME samples and SD10; B) SME samples and SD10; C) HVO samples and SD10.
