# Supplementary material for: The Toxic Effects of Petroleum Diesel, Biodiesel, and Renewable Diesel Exhaust Particles on Human Alveolar Epithelial Cells
Source: J Xenobiot. 2024 Oct 9;14(4):1432–49. doi: 10.3390/jox14040080 (PMC11503417; doi:10.3390/jox14040080)

Supplementary file S7. Cell cycle results.

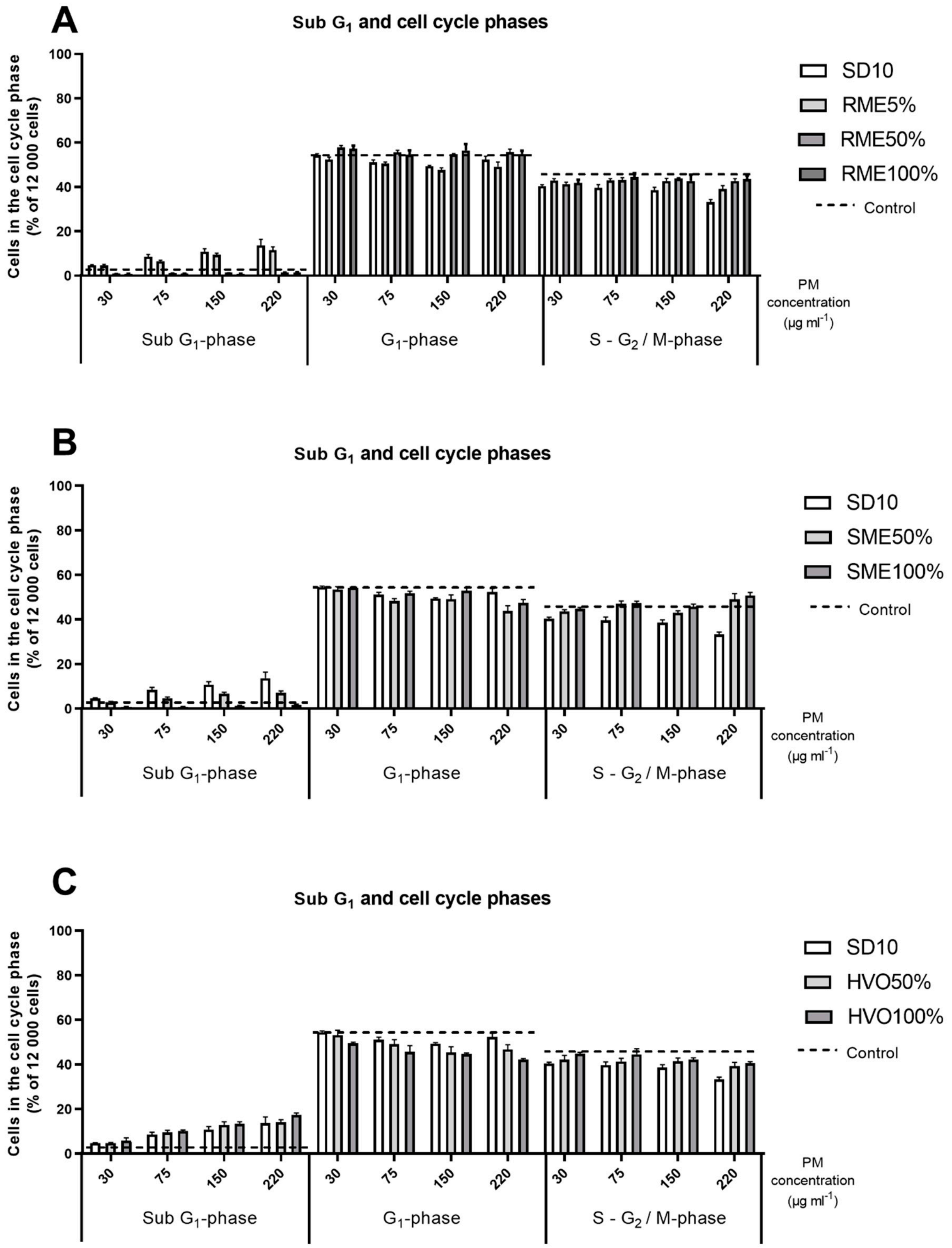

Cells in different phases of cell cycle after 24 h exposure to PM emission samples from biodiesels and SD10 diesel. Bars represent four concentrations (30, 75, 150, and 220 mg ml^{-1}); whiskers are the standard error of mean (SEM). A) RME samples and SD10; B) SME samples and SD10; C) HVO samples and SD10.

Gating strategy for cell cycle analysis:

First, we created a gate based on forward scatter (FSC) and side scatter (SSC) to identify live cells and to gate away dead cells and obvious debris. Next, we gated single cells using a plot of pulse height vs. pulse area to exclude doublets and aggregates. Then we combined these gates and applied them to the PI histogram plot to ensure that only the desired cell population was analyzed. Finally, we analyzed the DNA content using the PI histogram, which shows different peaks corresponding to the G0/G1, S, and G2/M phases of the cell cycle.

1. Gating cells from debris

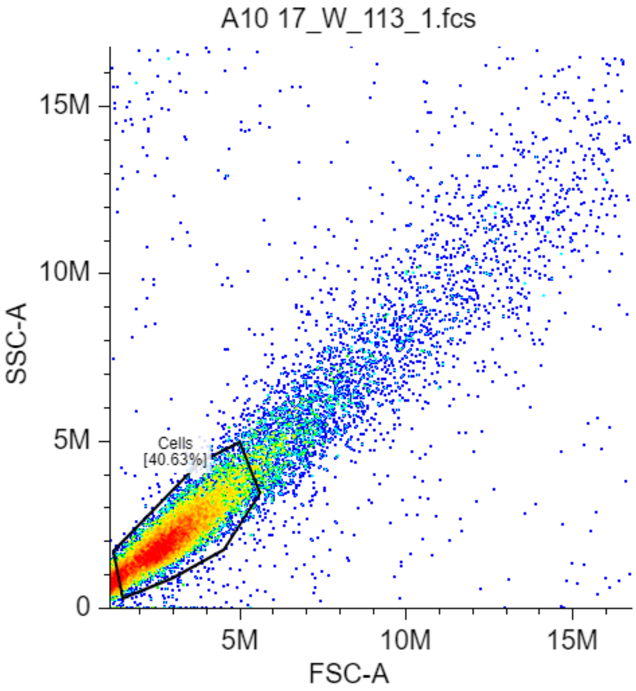

2. Gating singlets

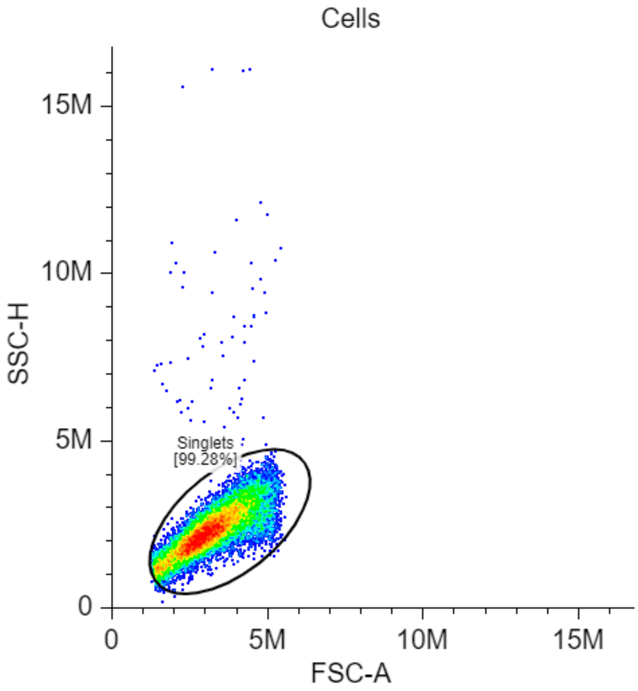

3. Cell cycle analysis

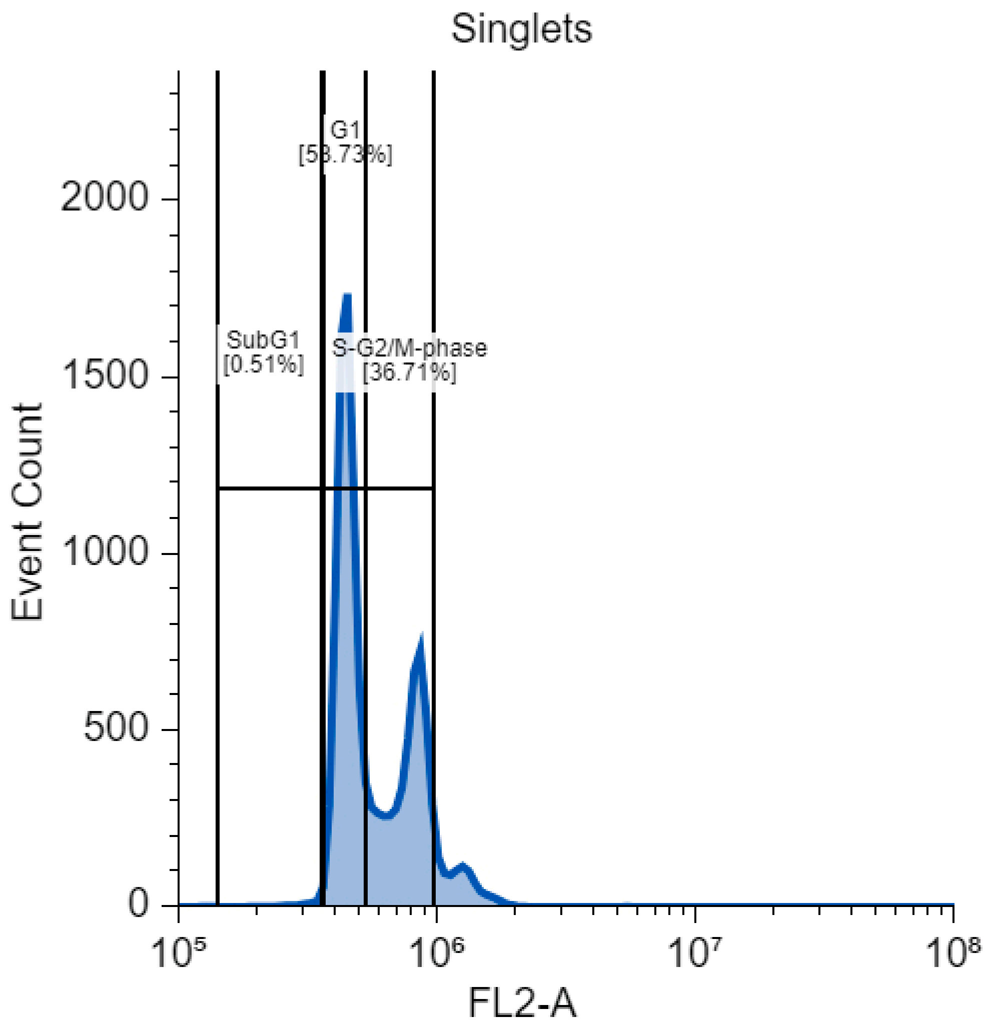

Supplement: Supplementary file 1 [file jox-14-00080-s001.zip › Supplementary file S7_Cell cycle.pdf]
