# Supplementary material for: The Toxic Effects of Petroleum Diesel, Biodiesel, and Renewable Diesel Exhaust Particles on Human Alveolar Epithelial Cells
Source: J Xenobiot. 2024 Oct 9;14(4):1432–49. doi: 10.3390/jox14040080 (PMC11503417; doi:10.3390/jox14040080)
Supplement: Supplementary file 1 [file jox-14-00080-s001.zip › Supplementary file S8_Inflammation.pdf]

Supplementary file S8. IL-6 and IL-8 results.

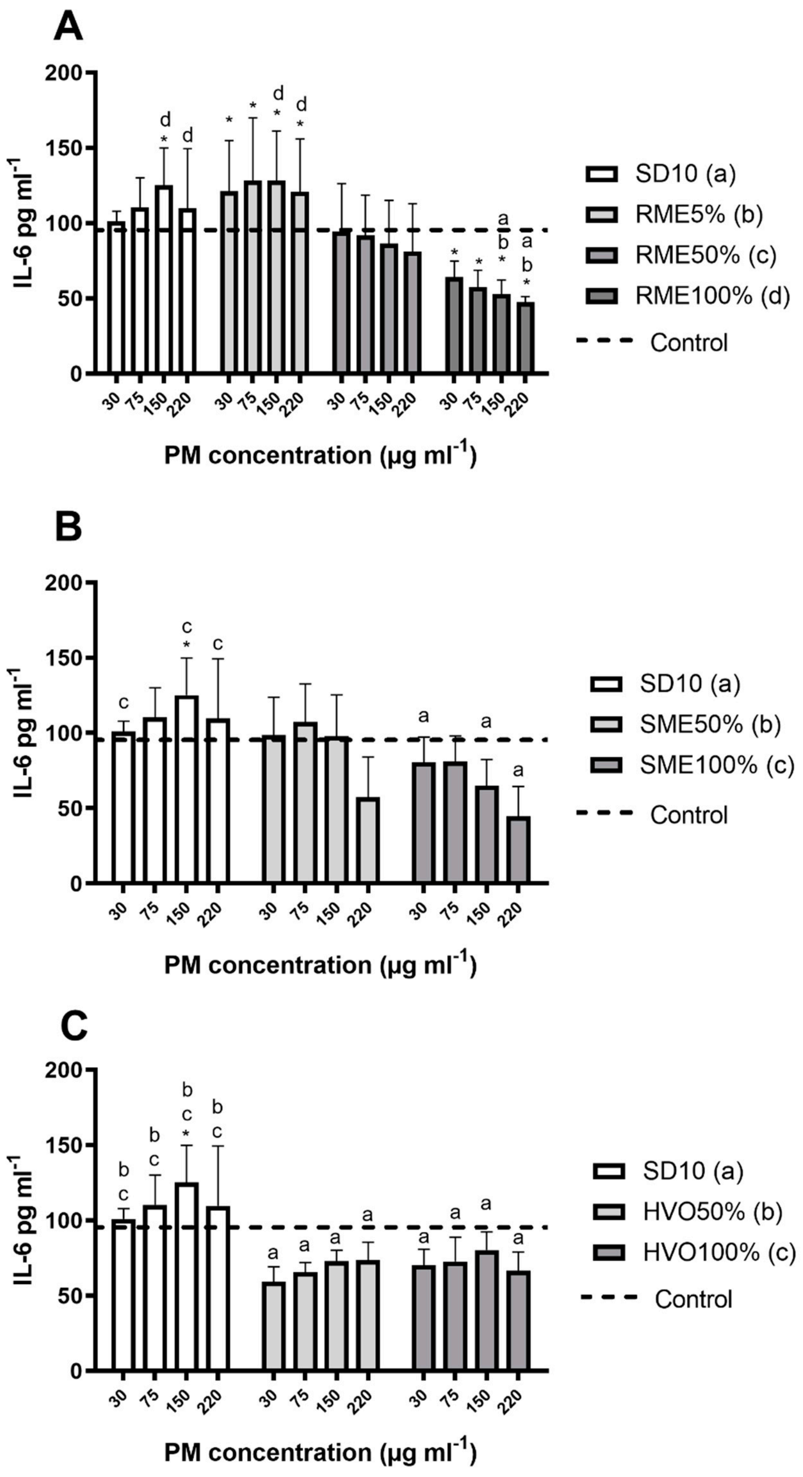

IL-6 concentration in cell culture medium after A549 cells exposure to PM emission particles from different biodiesels, renewable diesel, and SD10 diesel (24 h). Bars represent four concentrations (30, 75, 150, and 220 mg ml^{-1}); whiskers are the standard deviation (SD). The asterisks indicate statistical significance compared to the control ($p < 0.05$) analyzed using the nonparametric Mann–Whitney U test. The lowercase letters indicate a statistically significant response between samples ($p < 0.05$) based on a Mann–Whitney U test. A) RME samples and SD10; B) SME samples and SD10; C) HVO samples and SD10.

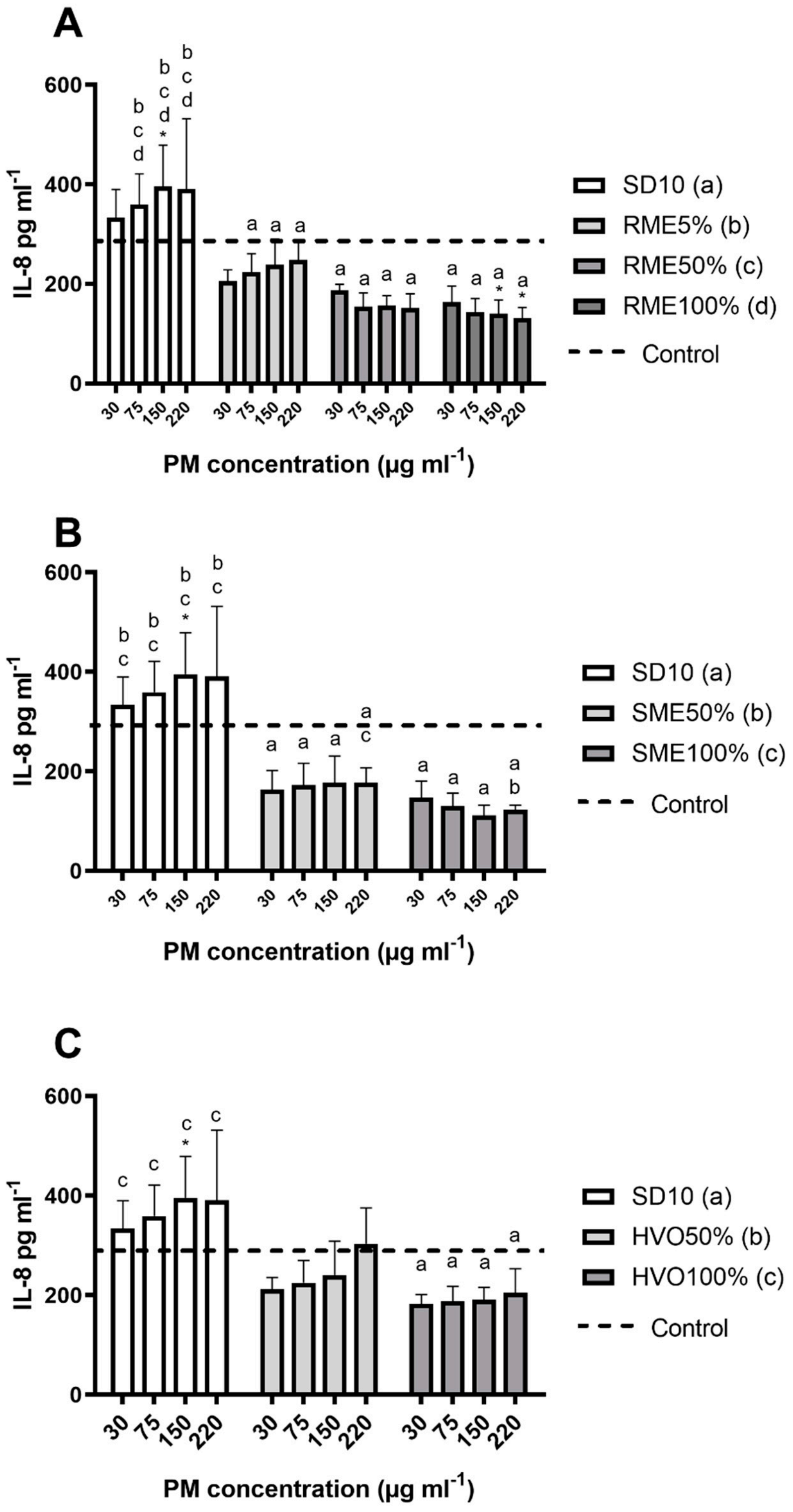

IL-8 concentration in cell culture medium after A549 cells exposure to PM emission particles from different biodiesels, renewable diesel, and SD10 diesel (24 h). Bars represent four concentrations (30, 75, 150, and 220 mg ml^{-1}); whiskers are the standard deviation (SD). The asterisks indicate statistical significance compared to the control ($p < 0.05$) analyzed using the nonparametric Mann–Whitney U test. The lowercase letters indicate a statistically significant response between samples ($p < 0.05$) based on a Mann–Whitney U test. A) RME samples and SD10; B) SME samples and SD10; C) HVO samples and SD10.
